# Peculiarities of social functioning in patients with negative symptoms in schizophrenia

**DOI:** 10.1192/j.eurpsy.2024.1589

**Published:** 2024-08-27

**Authors:** N. O. Maruta, Y. A. Kushnir, G. Y. Kalenska

**Affiliations:** Borderline Psychiatry, “Institute of Neurology, Psychiatry and Narcology of NAMS of Ukraine” SI, Kharkiv, Ukraine

## Abstract

**Introduction:**

The prevalence of schizophrenia in the world is between 0.4 and 1.4%, and the number of patients with negative symptoms (NS) in this group reaches 90%. NS are considered key components of schizophrenia that negatively affect social functioning (SF) and quality of life in patients with schizophrenia. The purpose of the study was to determine the features of SF among patients with NS in schizophrenia.

**Objectives:**

Features of SF in 252 patients with NS in schizophrenia (main group) and in 79 patients with positive symptoms (PS) in schizophrenia (comparison group) were examined.

**Methods:**

A set of methods was used: Scale of personal and social functioning (PSP), which is a semi-structured interview and allows to assess the social status of patients, their functioning and satisfaction with the relevant field and statistical methods.

**Results:**

The analysis of the social and personal functioning of patients was carried out in four domains: socially useful activities, personal and social relationships, attention to oneself and one’s condition, restless and aggressive behavior patterns. In the sphere of socially useful activities, including work and study, in a significant part of patients with NS in schizophrenia, SF violations were expressed at moderate (41.27 ± 1.26) % and significant (33.33 ± 1.08) % levels. In the sphere of personal and social interaction, 41.27 % of patients had significant violations, 28.97% of patients had moderate violations, and 21.83% had severe violations in the social sphere. In the field of self-care, 21.83% of patients had no violations, in 36.90% - violations in self-care were weakly expressed, and in 26.19% of people - moderately expressed.

When comparing the obtained results with patients with PS in schizophrenia, it was established that among patients with NS in schizophrenia there were more patients with significant impairments in the sphere of social activity (33.33%, p = 0.033, DC = 1.42, MI = 0, 07).. Patients with NS in schizophrenia were distinguished by a greater number of patients with significant impairments in the sphere of social interaction (41.27%, p = 0.001, DC = 2.58, MI = 0.24).. In the field of self-care, there were more persons with no violations among patients with NS in schizophrenia (21.83%, p = 0.008, DC = 3.33, MI = 0.20). There were more patients with the absence and weak expression of aggressive behavior patterns among patients with NS in schizophrenia (30.95%, p = 0.0001, DC = 10.87, MI = 1.55 and 45.63%, p = 0, 0001, DC = 6.54, MI = 1.16, respectively) in comparison with patients with PS in schizophrenia.

**Conclusions:**

The obtained data should be taken into account when creating psychocorrective programs for patients with NS in schizophrenia.

**Disclosure of Interest:**

None Declared

